# The mode of interfragmentary movement affects bone formation and revascularization after callus distraction

**DOI:** 10.1371/journal.pone.0202702

**Published:** 2018-08-23

**Authors:** Lutz Claes, Nicholaus Meyers, Julian Schülke, Sandra Reitmaier, Svenja Klose, Anita Ignatius

**Affiliations:** Institute of Orthopedic Research and Biomechanics, Center of Musculoskeletal Research, University of Ulm, Ulm, Germany; University of Zaragoza, SPAIN

## Abstract

Callus distraction is sometimes associated with a delay in the maturation process and serious complications. It is believed that these complications are often caused by instability of the bone segment fixation. Typical fixation devices, such as ring-fixators, show significant deformations in all directions under external loading and muscle forces. This leads to axial compression and tension as well as shear movements in the healing area. Herein we investigated the hypothesis that the direction of interfragmentary movement after callus distraction affects the bone formation and revascularization during the maturation process. Two custom fixator systems were designed to apply a protocol of lateral callus distraction and subsequent cyclic stimulation of the regenerate tissue. One fixator system was used to apply either compressive or tensile stimulation while the other was used to apply shearing stimulation. The fixators were applied to the tibial surface of the right hind leg of sheep specimens. During lateral callus distraction, a titanium plate was elevated by 0.275 mm perpendicular to the long axis of the bone twice daily, resulting in a 5.5 mm gap at the end of the ten-day distraction phase. Following a seven-day consolidation phase, the regenerate in the gap between tibial cortex and titanium plate was stimulated once daily by cyclic movement for 120 cycles. The stimulation was applied for 18 days with amplitudes of 0.6 mm in compression (Group C) or tension (Group T), or a 1.0 mm shear amplitude (Group S). Seven weeks postoperatively the specimens were analyzed for quantity of bone formation, the presence of cartilage and fibrous tissue, and blood vessel density. There was a significantly higher blood vessel density (4.6 ± 1.6%) in Group C than in Group T (1.2 ± 0.4%) or Group S (1.0 ± 0.5%) (p < 0.01). The amount of bone was significantly higher in Group C (25.6% ± 13.0%) than in Group T (13.5 ± 4.9%) (p < 0.05). Group S showed a similar amount of bone (14.0 ± 10.7%) to Group T. The results show that bone formation and revascularization are dependent on the direction of interfragmentary movement and that the cyclic compression best stimulates the healing process.

## Introduction

The method of callus distraction is a well-accepted treatment for the correction of skeletal defects [1]. Unfortunately, the newly-formed bone after the distraction process has finished is very weak and requires approximately twice as much time as the distraction to mature to a strength that allows the removal of the fixation device [1]. It is during this long maturation phase that most of the complications occur and it is believed that they are often caused by instability of the bone segment fixation [1, 2]. Typical fixation devices, such as ring-fixator and hybrid systems, show significant deformations in all directions under external loading and muscle forces of the operated extremity [3]. This leads to axial compression and tension as well as shear movements in the callus healing area [4]. Often, these complex interfragmentary movements (IFM) lead to a delay in the maturation process and serious complications [1, 5]. Ilizarov noted that translational shearing movements perpendicular to the bone axis, in the plane of the osteotomy or corticotomy, are particularly hindering to bone formation [1].

The maturation phase of the callus distraction procedure is biomechanically similar to a fracture healing situation; the gap between the two bone surfaces experiences three-dimensional IFM due to an elastic fixation and physiological loading. In the absence of callus distraction studies on the effect of different movement directions on bone healing it is therefore interesting to analyze the IFM and revascularization in fracture healing studies.

Ilizarov’s observation [1] regarding the critical effects of shear movements is supported by a previous experimental fracture healing study performed by our group that compared cyclic, 1.5 mm, predominantly axial movement in one group with 1.5 mm, predominantly translational shear movement in a second group of tibia osteotomies in sheep [6]. We found that bone formation was considerably reduced and bone healing delayed in the shear group compared to the axial group. Other studies did not show a negative effect of shear movement compared to axial movement. The experimental conditions, however, were different and incomparable.

As early as 1960, Pauwels [7] mentioned that a fracture shows different tissue developments on the tension and compression side of the fracture healing zone under bending loads. He found greater callus formation with predominantly endochondral ossification on the compression side and smaller callus formation with mainly connective tissue on the tension side. These results are supported by an experimental study of Hente and colleagues [8]. A cyclic bending stimulation of an osteotomy of the sheep tibia stimulated up to 25 times larger periosteal callus formation on the compression compared to the distraction side.

One other possible cause for the delayed healing under critical IFM is the reduced revascularization in the bone healing area. There are, however, no studies known that investigated the effect of IFM in specific directions on the bone regeneration and revascularization after callus distraction.

There are only a few fracture healing studies designed to have an IFM dominant in one direction with diminutive IFM in the remaining directions that investigated the bone healing and revascularization. Previously, we studied the bone healing in a sheep osteotomy with a custom ring fixator under controlled axial compression with only minor IFM in the other directions [9]. After nine weeks, we found that the initial axial IFM of 1.0 mm in one group led to a significant increase in fibrocartilage, a decrease in bone formation, and an approximate 20% reduction of revascularization in the osteotomy gap compared to another group of 0.2 mm IFM.

Lienau et al. [10] investigated bone healing in a similar sheep osteotomy model stabilized with two different unilateral external fixators. Their study initially allowed larger translational shear IFM than axial compression under loading. They found that an increase in the initial translational shear movement from 0.52 mm in one experimental group to 0.78 mm in another group led to a decrease in mineralized bone and an increase of fibrous tissue after six weeks and an approximately 45% decrease in revascularization of the osteotomy gap after nine weeks.

This indicates that larger IFM had similar effects on tissue differentiation but different effects on revascularization in both studies. Fivefold higher axial compression decreased revascularization by only 20% whereas the relatively small increase of shear movement (50%) led to a decrease in revascularization of 45%, indicating a particular sensitivity of revascularization to shear movements. This is in accordance with early observations of Rhinelander [11] who found that blood vessel growth in unstable fractures with dislocations of the fragments is hindered at the level of the fracture due to shear movements.

To study the effect of isolated IFM directions on bone formation and revascularization during the maturation process, novel callus distraction devices were developed. This allows the isolated application of compressive, tensile, and shearing movement [12] after callus distraction. The hypothesis of this study is that the direction of IFM after callus distraction affects the bone formation and revascularization during the maturation process of the newly formed bone.

## Materials and methods

### Study design

A custom-designed system, which allowed a protocol of lateral callus distraction and subsequent cyclic compressive, tensile, or shearing stimulation of the regenerate tissue [12] was applied in an ovine model to the anteromedial tibial surface of the right hind leg.

For the animal experiment, 18 adult female Merino sheep (age: 4–6 years; weight: 75–100 kilograms) were operated in series of six and at the time of surgery, randomly distributed in three groups of differing stimulation directions. After lateral callus distraction, 120 cyclic movements with an amplitude of 0.6 mm in compression (Group C) or tension (Group T), or a 1.0 mm shear amplitude (Group S) were applied. The animals were sacrificed seven weeks postoperatively and the specimens analyzed for quantity of bone formation, presence of other tissue types, and blood vessel density.

All interventions on living animals were performed according to the regulations of EU Directive 2010/63/EU for animal experiments and conformed to ARRIVE guidelines (Animal Research: Reporting of in Vivo Experiments). The experiments were approved by the local ethics committee (Registration-no. 1168, Regierungspräsidium Tübingen, Germany).

### Surgery

**Fig 1:**
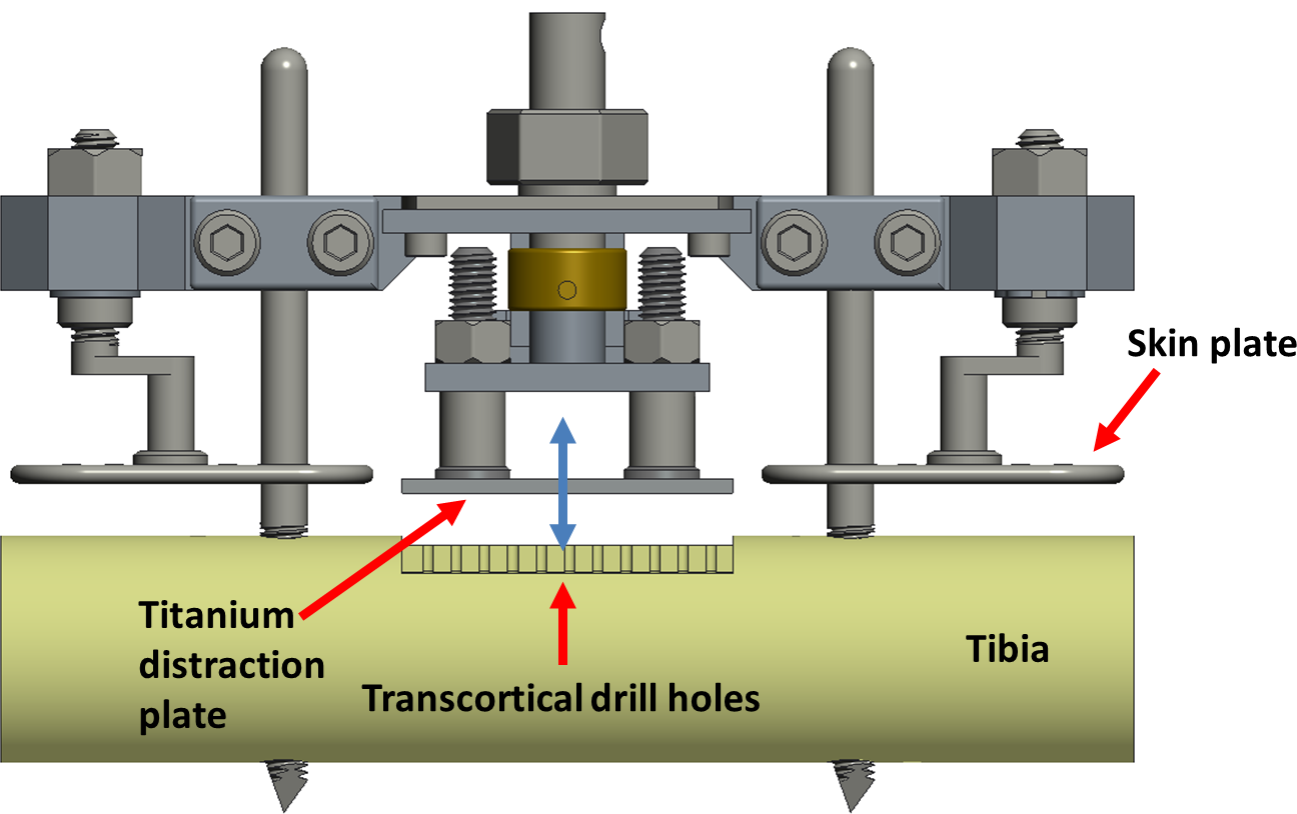
System for the lateral callus-distraction and subsequent cyclic compression and tension stimulation of the newly formed bone by the titanium plate. A modified system was used for shear movements described in detail in a previous publication [12].

**Fig 2:**
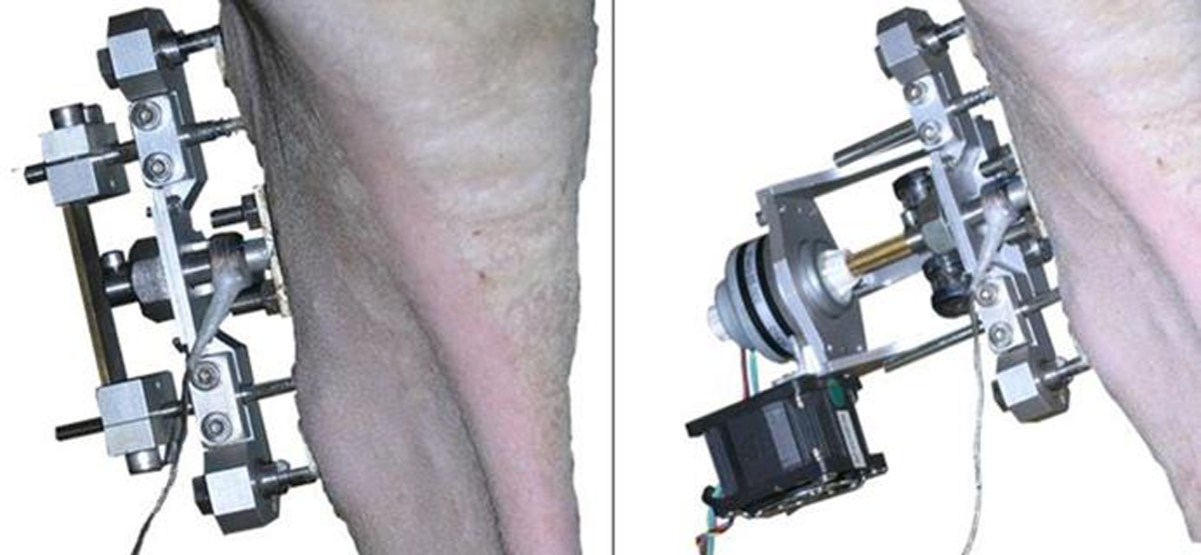
Callus distraction system applied to the sheep tibia. Left) after callus distraction, Right) with stepper motor applied to produce cyclic compression and tensile movements of the titanium plate.

The surgical treatment followed the protocol published previously [13]. In brief, the skin of the anteromedial aspect of the right tibia was incised and prepared to visualize the bone surface. Two Schanz screws (DePuy Synthes Companies, Zuchwil, Switzerland), Ø 5 mm proximally and Ø 4 mm distally, were inserted in a standardized position 55 mm apart, perpendicular to the long axis of the bone. The periosteum was then resected in a defined rectangular field between the Schanz screws and a flat plane was milled into the cortex, approximately 0.5 mm in depth (Fig 1). The removed periosteum and milled plane were created to complement the dimensions of the titanium plate (35 x 10 mm). Subsequently, 22 holes, 1.1 mm in diameter were drilled in two parallel rows, 3 mm apart, into the medullary cavity underneath the prepared site to allow sufficient neovascularization of the regenerating tissue. The titanium plate was placed on the cortical surface and the surgical site was closed. The distraction device was then mounted to the Schanz screws with the titanium plate held in position by a threaded connection to a load cell and shaft coupling in series (Fig 2).

### Post-operative protocol

Following surgery, the animals were housed on straw bedding with water and hay provided *ad libitum*. A veterinarian assessed the animals’ health daily and regularly cleaned and redressed the surgical wound. The experiment spanned 49 days, divided into a ten day post-surgical latency period, ten days of twice-daily distraction, seven days consolidation with no motion at the titanium plate, 18 days of cyclic stimulation, and a final three day rest period before euthanasia.

During the distraction phase, the titanium plate was elevated by 0.275 mm perpendicular to the long axis of the bone twice daily, resulting in a 5.5 mm gap at the end of 10-day distraction phase. The mechanical anchorage of the granulation tissue growing in between the porous hydroxyapatite coated surface of the titanium plate allows the transfer of tensile and shearing forces from the plate to the biological tissue between the plate and bone surface. A seven-day consolidation phase with no motion at the titanium plate followed distraction. Beginning on day 28 after surgery, the regenerate tissue between the tibial cortex and titanium plate was stimulated once daily by cyclic movement of the distraction plate using a removable stepper motor/linear actuator (Haydon Kerk Motion Solutions, Waterbury, CT, USA).

In detail, both Group C (compression) and Group T (tension) were stimulated with 0.6 mm amplitude perpendicular to the bone surface at a frequency of 0.33 Hz for six minutes, resulting in 120 cycles. Group S (shear) was stimulated with 1 mm movement parallel to the bone surface at a frequency of 0.2 Hz for ten minutes resulting in 120 cycles. Because the strain rate may have a more pronounced effect on the bone formation than the loading frequency within the range applied in this experiment, we kept the initial strain rate (plate velocity, 0.4 mm/s) between groups of different amplitudes constant and controlled the total number of cycles. The amplitudes of movements were selected based on a numerical model of the experiment used to calculate the distortional strain and hydrostatic pressure in the initial mesenchymal tissue [14] after the distraction phase. The amplitudes used in each group were predicted to induce a similar magnitude of hydrostatic pressure (0.15 MPa) and a distortional strain > 15%. Based on experimental fracture healing studies, [6, 15, 16] and the tissue transformation hypothesis for fracture healing, these biomechanical conditions should lead to endochondral ossification and fibrous tissue development [17].

### Preparation

After sacrifice, the right tibia was explanted and the distraction device detached. The region of interest (ROI) was cut between the two rows of transcortical boreholes leaving one slice for radiological analysis and one for histological analysis.

### Radiography

**Fig 3:**
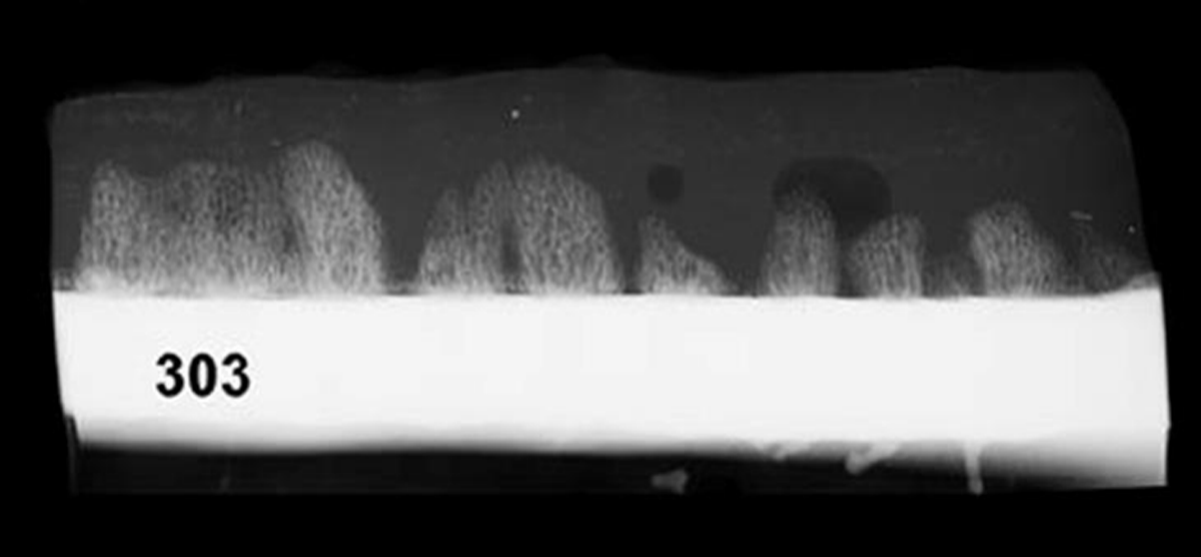
X-ray of a 3 mm longitudinal section through the bone formation zone. The newly formed bone spicules grow above the cortex of the sheep tibia at the location of the boreholes. The spicule heights are different although the mechanical environment is the similar along the cortex. The titanium plate was removed before this X-ray was taken and was located above the spicule.

One 3 mm thick longitudinal section through the area of interest of each sheep was radiologically investigated using an X-ray machine (MX-20, Faxitron Bioptics, 25 kV, 19 sec.). This allowed a first qualitative analysis of the new bone formation above the cortical surface (Fig 3).

### Histology

Undecalcified histological sections were prepared by embedding formalin-fixed specimens in polymethyl methacrylate (PMMA, Roth GmbH, Karlsruhe, Germany). The ground sections (70 μm) were stained with Paragon (Waldeck GmbH, Münster, Germany) and evaluated using light microscopy (Leica DMI6000B, Leica microsystems, Wetzlar, Germany) at each available magnification between 12.5–400 x. The regenerate tissue was quantitatively assessed for tissue types. The tissue parameters investigated were bone including osteoid (B), fibrous tissue (Fb), fat tissue (Fa), and vessels (V). Cellular morphology was studied to determine the state of differentiation and metabolic activity. Histomorphometry was performed using point-counting analysis. A 12x3 mm^2^ (width x height) area in the middle of the regenerate was scanned with a grid. The grid fields (12 x 3) with 100 count points each provided 3600 analysis points, sufficient to present the relative values of tissue parameters investigated with < 5% error [18]. The amount of blood vessels and tissues in the ROI was described in percent. Furthermore, the height of the conic osseous structures (spicules) above the medullary boreholes was measured using DataViewer (Bruker, Billerica, Massachusetts, USA) and the mean height of spicules for each specimen were calculated.

### Statistics

Histomorphometry was compared using the Kruskal-Wallis test for non-parametric data due to the number of specimens barring normal distribution. The level of significance was set at α = 0.05.

## Results

### Surgery and postoperative protocol

Surgeries were performed successfully with animals recovering to symmetrical weight bearing after about three days. The manipulations during distraction and cyclic stimulation were well tolerated. The sheep showed no marked signs of pain or discomfort during manipulations. The data of Group C were already published previously and served as a basis for comparison in this publication [19].

### Radiography

The X-rays showed new bone formation in columns (spicules) above the transcortical boreholes (Fig 3). Although the applied distraction and the mechanical stimulation during the maturation phase was constant along the chain of boreholes, the height of the spicules showed considerable differences.

### Histology

**Fig 4:**
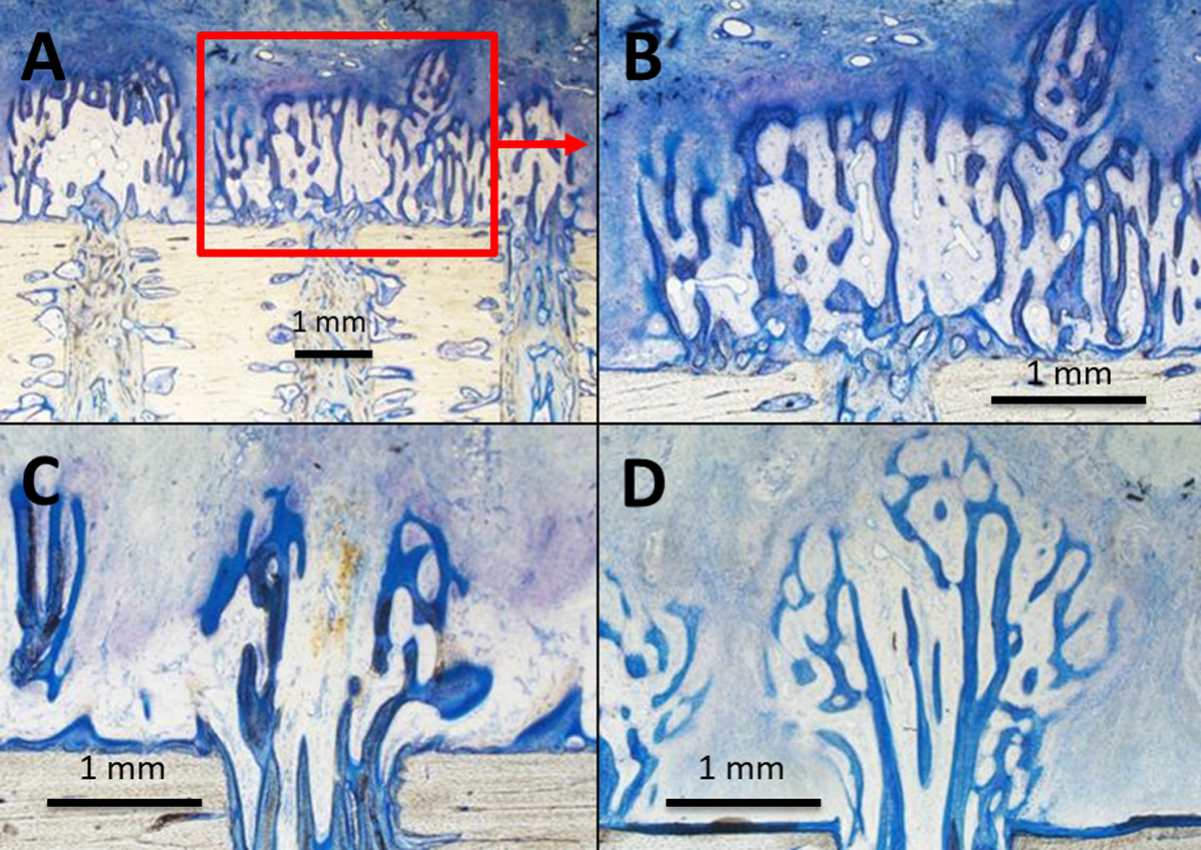
Longitudinal sections through the bone formation zone above the boreholes, Paragon staining. A) Group C, magnification 12.5x, three boreholes with newly formed trabecular bone formation. B) Group C, higher magnification (25x) of Fig 4A, large bone formation with high amount of vessels. C) Group S and D) Group T, less bone formation and lower vessel density (magnification 25x). Cortical bone (at the bottom) is stained beige, newly formed trabecular bone dark blue and amorphous granulation tissue light blue to blue. Vessels are not specifically stained and are white in its lumen.

**Fig 5:**
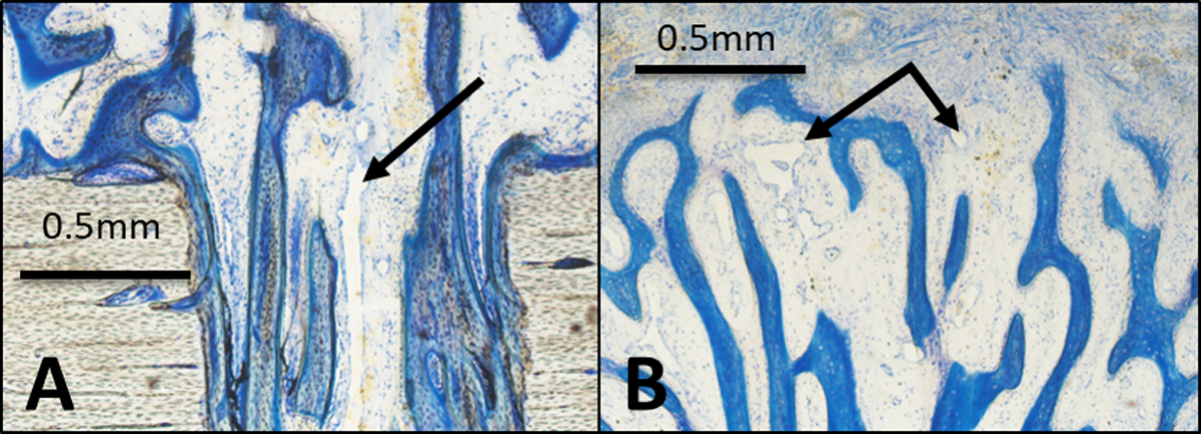
A) Exit of the borehole: lamellar bone formation inside the borehole channel with one central large vessel (arrow) connecting the medullary space with the new bone formation at the surface of the tibia. B) Blood vessels at the top of the spicule (arrow, magnification 50x), Paragon staining.

Histology showed no qualitative differences regarding tissue and cell differentiation between the groups (Fig 4). The regenerate was composed of woven bone in progressive maturity states that had grown from the base of the transcortical boreholes towards the titanium plate (Fig 4). Seams of osteoblasts covered the bony trabeculae (Fig 4). There was a layer of organized collagen fiber bundles and fibroblast-like cells covering the spicules reminiscent of mesenchymal condensations. The remainder of the gap was filled with vascularized fibrous tissue (Fig 4). Ossification was purely intramembranous; there were neither morphological signs of terminally differentiated chondrocytes nor endochondral ossification. Neovascularization mainly originated from the medullary cavity and ran through the boreholes, branching out in the spicules (Fig 5).

**Fig 6:**
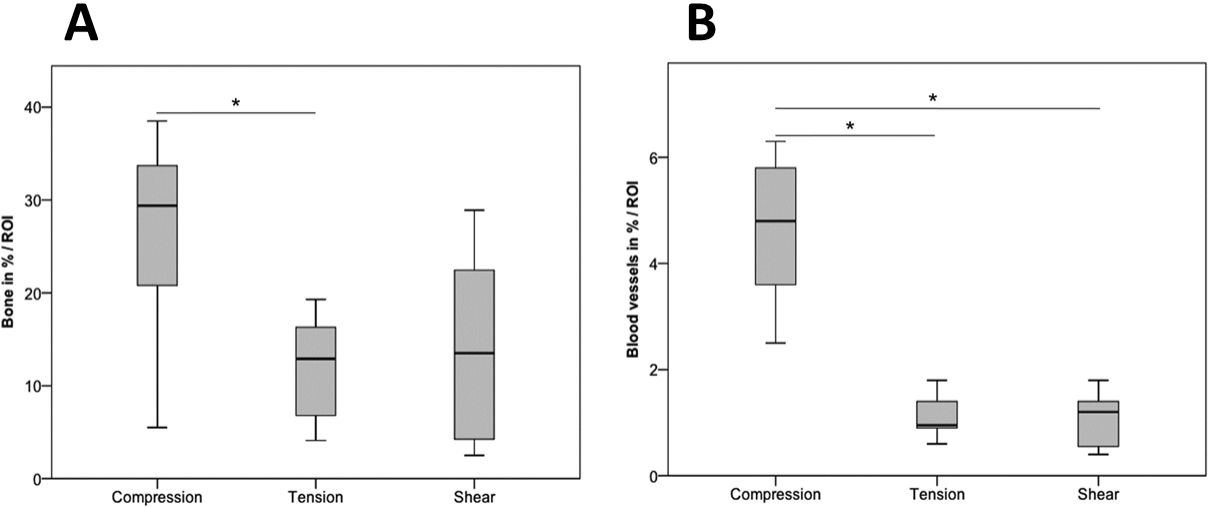
A) New bone formation above the tibia surface after cyclic compression, tensile and shear stimulation (* p<0.05). B) Blood vessel density in the newly formed bone after cyclic compression, tensile and shear stimulation (* p<0.01). The compression data are already published in [19] and shown for comparison.

There was a significantly higher blood vessel density (4.6 ± 1.6%) in Group C (Figs 4B and 6B) than in Group T (1.2 ± 0.4%, Fig 4D) or Group S (1.0 ± 0.5%, Fig 4C) (p < 0.01). The amount of bone in the ROI was significantly higher in Group C (25.6% ± 13.0%, Figs 4B and 6A) than in Group T (13.6 ± 4.9%, Fig 4C) (p < 0.05). Group S showed a similar amount of bone (14.0 ± 10.7%, Fig 4D) to Group T. Group T and Group S showed the highest amount of fibrous tissue (both about 85% whereas Group C exhibited lower values of about 70%). The amount of fat was very low (< 0.3%) for all groups. The mean bone spicule height reached different levels with 2.8 ± 1.5 mm in Group C compared to 1.8 ± 0.8 mm in Group S and 1.5 ± 0.7 mm in Group T.

## Discussion

The hypothesis that the direction of cyclic IFM after callus distraction affects the bone formation and revascularization during the maturation process of the newly formed bone was confirmed. The group with cyclic compression showed significantly more bone formation and more vessels than the group with tensile or shear stimulation.

It is known that moderate, cyclic, predominantly compressive stimulation during or after callus distraction can accelerate the bone healing process [20–22]. In an ovine, segmental bone transport model, we previously found that a cyclic stimulation of 0.5 mm, predominantly compressive interfragmentary movement during the maturation phase significantly enhanced bone healing in comparison to stiff fixation, or either 1.2 mm or 3.0 mm of cyclic axial movement [20]. Leung et al. performed a callus distraction study where 12 goats were allowed to bear weight immediately and 12 were not allowed to bear weight. The study showed more bone formation in the weight bearing group confirming the beneficial effect of cyclic, predominantly compressive movements in the healing zone [22]. Kassis et al. investigated bilateral callus distraction in rabbits [21]. One limb was stimulated by axial micromovements after the distraction procedure (450 load cycles per day, 0.188 mm amplitude). The stimulated limb showed significantly more bone formation indicating the beneficial effect of compressive stimulation to bone healing.

Similar effects are seen in fracture healing studies where cyclic, axial compression amplitudes stimulated callus healing [6, 15, 23]. For the maturation process after callus distraction as well as for fracture healing processes with fracture gap sizes of 2–3 mm, an initial compressive interfragmentary strain up to 50% seems to be stimulatory for endochondral bone formation [6, 15, 16, 23].

The compressive strain resulting from the chosen cyclic movement in this lateral callus distraction study falls within this strain range (> 16% at the beginning and < 50% at the end of stimulation). Therefore, it is not surprising that under this 0.6 mm cyclic compression significantly more bone was formed than under 0.1 mm movement as shown in our previous study [19]. Under the very small cyclic compressive movement (0.1 mm), comparable with a very stable fixation during callus distraction and maturation (< 5% interfragmentary strain), only a small amount of intramembranous bone was formed in the same experimental model [19]. Compared to this very small compressive stimulation (0.1 mm, < 5% strain), the tissue strain stimulation with larger purely tensile and shear movement in this study (> 16%, < 50%) also increased the bone formation during the maturation phase. However the bone formation did not reach the level of the large compression group (0.6 mm). One possible explanation for this difference is the density of vessels in the healing area that seems to be influenced by the direction of the IFM.

From fracture healing studies, it is known that the tensile side of a fracture subjected to bending forms a smaller callus, mainly composed of fibrous tissues. In contrast, a larger bony callus forms through endochondral ossification on the compressive side [7]. An experimental study in sheep by Hente et al. [8] showed that a cyclic bending movement applied to a tibia osteotomy of sheep led to significantly more periosteal callus formation on the compressive than on the tensile side [8].

With the model described in this study, it could be shown for the first time that pure, cyclic tensile strain applied in the maturation phase after stable callus distraction reduces the bone formation relative to cyclic compression even though the magnitude of distortional strain was nearly identical to those under cyclic compression.

Similar to the effect of cyclic tensile strain we found significantly lower bone formation for shearing movement when compared with compressive movement. As previously mentioned, experiments under pure shearing movements had not yet been conducted for fracture or osteotomy models due to the elasticity of the fracture fixation devices and the three-dimensional loading of the extremities. Therefore only results from studies with predominantly shearing movements can be compared to studies with predominantly compressive movements. In a previous study, we compared cyclic, 1.5 mm, predominantly axial movement in one group with 1.5 mm, predominantly translational shear movement in a second group of tibia osteotomies in sheep [6]. We found that bone formation was considerably reduced and bone healing delayed in the shear group compared to the axial compression group.

The reduced bone formation found in fracture healing studies under predominantly shearing and tensile movements compared to predominantly compressive movements is in accordance with the results found in this study after callus distraction. The question remains as to why the different movement directions affect the bone formation even though the distortional strains are in the same range. One possible reason might be the capability for the revascularization under different directions of interfragmentary movements as vascularization is a prerequisite for bone formation.

There are no studies known that investigated the effect of IFM in specific modes on the revascularization after callus distraction. The main expression of angiogenic factors during callus distraction occurs during the distraction phase of the treatment [24] which was identical for all three groups in the present study. However, the quantity of vessels found in the maturation phase was significantly different for the different movement directions with the greatest vascularity occurring under cyclic compression.

The greater effects of larger shear movement to the reduced revascularization compared to larger compression movements were found in fracture healing studies where an approximately 500% increase in axial compressive amplitude decreased the revascularization by only 20% [9] whereas the much smaller increase of shear movement (50%) led to a more significant decrease in revascularization of 45% [10]. A study on fracture healing under stable and very unstable conditions in sheep investigated the effect of large predominantly shear movements to the regulation of blood vessel formation [25]. They found a significantly lower expression of genes for blood vessel formation in comparison to stable fixation. This indicates the particular sensitivity of revascularization to the direction of movements. By 1974 Rhinelander [11] had already mentioned that dislocation of fracture fragments (shear movement) disturbed the growth of vessels in the callus and delayed the bone healing process based on microangiographic images from fracture healing studies in dogs.

In our previous study using the same experimental model, it could be shown that the same vessel density results from both small and large amplitude cyclic compression, but the larger stimulation led to significantly more bone formation [19]. This indicates that the new bone formation under cyclic movement is driven primarily by the magnitude of tissue strain when sufficient perfusion is available. Given the same (Group T) or similar (Group S) amount of distortional strain as in the compression group, the vascularization of the mesenchymal tissue in the callus maturation zone seems to be correlated to the direction of stimulation. Under compression, the vascularization was about four times greater than under tension or shear. Correspondingly, bone formation was approximately twice as high in compression compared to tension or shear. This indicates that the reduced bone formation may be due to reduced vessel formation resulting from the specific mode of IFM.

Another clue for the effect of the vascularization on the bone formation is the difference in spicule heights resulting from the same mechanical conditions. Although the tissue strain during distraction and stimulation was very similar for all locations above the boreholes in each group, the spicule heights show considerable variation (Fig 3). The most reasonable explanation is that the perfusion of each spicule depends on the local vasculature available to the spicule’s respective transcortical borehole from the intramedullary space.

Another interesting observation was that only intramembranous bone formation and no endochondral ossification could be seen in the bone healing zone independent of the chosen direction of IFM. In callus distraction studies with osteotomies [5, 26–29] endochondral ossification or fibrocartilage was seen under interfragmentary movements in a similar range as in this study. One possible explanation for the purely intramembranous bone formation after stable callus distraction is that osteoblastic differentiation during the distraction period is very strong; only purely uniaxial tissue strain is applied and the influence persists during the maturation period when other mechanical stimuli are applied. This phenomenon was seen in our previous study with the same lateral callus distraction model where, after the distraction period, the bone healing front continued growing under stable conditions without any further mechanical stimulus [13]. It seems that if the callus distraction is performed with pure tensile strain, cell differentiation is strongly triggered in the osseous direction and can suppress differentiation to chondrocytes under tissue deformations that usually lead to chondrocyte differentiation and endochondral ossification in the distraction and maturation phases [1, 5, 20, 27].

Based on the results of this study, the best bone formation for callus distraction procedures can be expected when a very stable distraction phase allowing the application of purely uniaxial tensile strain is followed by a moderate cyclic axial compression during maturation due to weight bearing activity.

## Supporting information

S1 File Histomorphometry data. Histomorphometry measurements for compression, tension, and shear stimulation groups.
(XLSX)
